# Assessing Disparities in Inappropriate Outpatient Antibiotic Prescriptions in Tennessee

**DOI:** 10.3390/antibiotics14060569

**Published:** 2025-06-01

**Authors:** Katie A. Thure, Glodi Mutamba, Callyn M. Wren, Christopher D. Evans

**Affiliations:** Healthcare-Associated Infections and Antimicrobial Resistance, Tennessee Department of Health, Nashville, TN 37243, USA

**Keywords:** antibiotic stewardship, antibiotic use, outpatient, health equity

## Abstract

**Background/Objectives:** In 2022, over 200 million outpatient antibiotic prescriptions were written in the U.S., with 30% deemed unnecessary. Previous studies have shown that demographic factors, such as age, gender, and race, influence antibiotic prescribing patterns. However, few studies have examined how social determinants of health contribute to health inequities in antibiotic prescribing. This study aims to explore these disparities in Tennessee using IQVIA data. **Methods**: The Tennessee Department of Health conducted a cross-sectional study using the IQVIA LRx and Dx databases, linking prescription data to diagnoses from 2022. Antibiotic prescriptions were categorized into three tiers based on appropriateness. A multivariable logistic regression model assessed factors such as age, gender, insurance type, and social vulnerability index (SVI) on antibiotic prescribing patterns. **Results**: Of 2,874,505 prescriptions analyzed, 59.3% were classified as inappropriate (Tier 3). Female patients and children were less likely to receive inappropriate antibiotics. Patients in lower SVI areas, indicating less social disadvantage, had lower odds of receiving unnecessary prescriptions. Medicaid and Medicare Part D beneficiaries had higher odds of receiving inappropriate antibiotics compared to those with private insurance. **Conclusions**: This study highlights significant health disparities in outpatient antibiotic prescribing in Tennessee. Male patients, older adults, and individuals in socioeconomically vulnerable areas are more likely to receive inappropriate prescriptions. These findings stress the need for targeted public health interventions to reduce unnecessary antibiotic use and address underlying health inequities, ultimately improving healthcare outcomes and reducing antimicrobial resistance.

## 1. Introduction

In 2022, healthcare professionals in the outpatient setting prescribed more than 200 million antibiotics in the United States (U.S.) [1]. Previous reports from the Centers for Disease Control and Prevention (CDC) estimate that 30% of antibiotics prescribed in this setting do not warrant antibiotic use [2,3]. Additionally, studies show antibiotic prescribing is influenced by numerous factors including gender, race, and age [2,4,5,6]. Social determinants of health play a critical role in shaping prescribing practices and can significantly impact both the demand for antibiotics and the likelihood of receiving them appropriately. While studies have explored antibiotic use at population levels, few focus on the inequities found in these prescriptions, and even fewer capture measures of appropriateness in their analyses. Further studies are needed to assess and address these inequities in appropriate antibiotic prescribing.

In previous studies, patients were more likely to receive antibiotics if they were female, resided in the South, and reported inability to afford prescription drugs [7]. In 2023, Kim et al. conducted a scoping review to characterize inequities in antibiotic prescribing within the United States [8]. Their review showed that out of 61 articles exploring the relationship between antibiotic prescribing and health equity, almost half were published between 2018 and 2021, and the vast majority assessed antibiotic prescribing from the outpatient setting. Studies included in their review established individual patient factors associated with increased antibiotic prescribing. For example, older adults, defined as age > 65, and younger children, defined as age < 10, were more likely to receive antibiotics for conditions such as viral respiratory illnesses [9,10]. Female patients were also more likely to be prescribed antibiotics than male patients [11]. Black patients were prescribed antibiotics less frequently than white patients [5,12]. Lastly, more antibiotics were consistently prescribed in the Southern region of the United States than in other regions [13]. Although the number of antibiotics prescribed does not directly correlate with the quality of care, it is important to be judicious about prescribing antibiotics appropriately.

To identify health inequities, including age, gender, socioeconomic status, and insurance, in the outpatient setting, the Tennessee Department of Health (TDH) evaluated the appropriateness of outpatient antibiotic prescriptions using a proprietary database. To our knowledge, this is the first study that utilizes IQVIA data, a large ambulatory prescription database, to understand these disparities in the context of appropriateness of antibiotic prescribing. Identifying populations that are inappropriately prescribed antibiotics is imperative to address health inequities that are pervasive in marginalized communities.

## 2. Results

There were 2,874,505 prescriptions in the LRx database that were able to be linked to diagnoses in the DX database. Tier 1 and Tier 2 diagnoses represented 13.4% (n = 385,008) and 27.3% (n = 784,020), respectively, of the cases. Tier 3 diagnoses accounted for 59.3% (n = 1,705,477) (Table 1). These tiers were further analyzed based on demographics, socioeconomic status, and insurance type.

Female patients comprised 61.8% of the overall antibiotic prescriptions, with more representation in Tier 3 (59.0%) when compared to Tier 1 and Tier 2. Older adults, particularly those aged 65 years and above made up more Tier 3 prescriptions (68.7%) than Tier 1 (16.7%) and Tier 2 (14.5%). In contrast, younger age groups, such as those aged 0–2 years had mostly antibiotics in encounters for Tier 2 diagnoses (60.6%).

Patients from lower SVI categories exhibited higher representation, with 823,019 individuals classified as low SVI and 697,206 as low-medium SVI. Within these groups, 13.7% and 13.2% of patients, respectively, were categorized under Tier 1 diagnoses, while 28.0% and 27.1% fell into Tier 2. Patients in the high SVI category comprised 703,704 total antibiotics, had mostly Tier 3 diagnoses (59.6%).

Female patients had 11% lower odds of receiving an antibiotic for a Tier 3 diagnosis compared to male patients, who served as the reference group (OR: 0.89; 95% CI: 0.89–0.89) (Table 2). Age influenced prescribing patterns, with young adults aged 20–29 years having the lowest odds of receiving antibiotics for indications that do not require therapy (OR: 0.26; 95% CI: 0.26–0.26), followed closely by children aged 0–2 years (OR: 0.27; 95% CI: 0.26–0.27) when compared to individuals aged 65 and older (reference group). However, these odds increased progressively with age, with adults aged 30–39 years (OR: 0.60; 95% CI: 0.60–0.61) and 40–64 years (OR: 0.87; 95% CI: 0.86–0.87) showing higher odds, but still lower relative to the reference group. SVI demonstrated minimal but statistically significant associations with antibiotic prescribing between the most and least vulnerable SVI categories. Compared to individuals in the high-SVI category (reference group), those in the low-SVI group had 6% decreased odds of inappropriately receiving antibiotics (OR: 0.94; 95% CI: 0.94–0.95). Odds were equivalent for individuals in the low-medium (OR: 1.00; 95% CI: 0.99–1.00) and medium-high (OR: 1.00; 95% CI: 0.99–1.00) SVI groups.

Insurance type was also a significant predictor of whether someone was prescribed an antibiotic for indications that do not require therapy. Medicaid recipients (OR: 1.17; 95% CI: 1.16–1.18) and Medicare Part D beneficiaries (OR: 1.20; 95% CI: 1.20–1.21) exhibited 17% and 20% higher odds, respectively, compared to privately insured individuals.

## 3. Discussion

The results of this current study begin to explore specifically an important gap in the current literature: health disparities in inappropriate, and in this case, unnecessary outpatient antibiotic prescribing. It highlights demographic and socioeconomic factors associated with increased likelihood of receiving an inappropriate antibiotic prescription.

In this analysis, female patients received more antibiotics, like previous studies, but they had an 11% lower chance of receiving antibiotics for conditions where they were not warranted, compared to male patients. This pattern points to the need for more awareness and training among healthcare providers to ensure that antibiotic prescriptions are based on clear clinical evidence, such as urinalysis with culture and antibiotic sensitivity results. Another key demographic with disparity in antibiotic prescribing was age. Children were less likely to be prescribed antibiotics for conditions where antibiotics are not indicated when compared to older adults.

Social determinants of health, including socioeconomic status, community vulnerability, and insurance type, significantly influence antibiotic prescribing practices. It is important to note that while these results are statistically significant, each odds ratio approached close to 1, suggesting there might be no clinical significance. Nevertheless, patients living in areas with lower SVI scores, indicating less social disadvantage, had lower odds of receiving antibiotics for conditions that do not require them when compared to areas with higher SVI scores. This suggests that when there are more opportunities to access consistent healthcare, there might be more access to proper diagnostics which help guide treatment. The results of this current study offer further exploration into previous studies that showed patients living in areas of greater socioeconomic deprivation had a lower likelihood of receiving a prescription [14]. This highlights the importance of addressing underlying social factors in public health initiatives, as improving access to care in vulnerable communities could lead to better health outcomes and more appropriate use of antibiotics.

Insurance type also played a significant factor influencing prescribing patterns. Patients with public insurance, such as Medicaid and Medicare Part D, were more likely to receive antibiotics for inappropriate conditions compared to those with private insurance. This might also highlight the limited access patients with public insurance face in interacting with regular preventive clinicians, such as primary care. Patients with public insurance might seek care in urgent care or emergency room settings where quick fixes, like antibiotics, are more likely to be prescribed. It also potentially highlights diagnostic limitations or may reflect differences in healthcare delivery, where private insurance programs may emphasize stricter adherence to diagnostic and/or treatment protocols. Conversely, individuals with private insurance might have more access to healthcare providers who may be more likely to prescribe antibiotics.

This study does have certain limitations that should be highlighted. IQVIA is a robust dataset; thus, it is possible that some results displayed significance, even for small differences. The data utilized in this study are based on ICD-10 codes. Although clinicians should be utilizing the correct code(s) for each visit, it is possible they were not specific enough. For example, if a provider had a high suspicion for bacterial infection but only included “fever” as the ICD-10 code at the time of the patient visit, it would have been deemed an inappropriate antibiotic prescription unless the ICD-10 code was eventually altered upon final diagnosis, a practice that occurs infrequently. Validation of ICD-10 codes at the individual practice level was beyond the scope of this analysis. Another limitation to this study is the Tier system utilized. For the purposes of this study, all antibiotics with a Tier 2 diagnostic code were considered appropriate. However, it is feasible that some of these would be considered inappropriate with more information provided. Given the lack of additional clinical information in the dataset, we were unable to fully determine if these antibiotics were truly appropriate and took a more lenient approach in coding them as “appropriate”. Finally, and unfortunately, data on patient race and ethnicity were not available in the IQVIA LRx/Dx database, meaning these demographics were unable to be included in the inappropriate analysis model.

The implications of these findings are significant for public health policy and healthcare practice. Leveraging the results of this study, TDH plans to develop public health resources to address these disparities to lower the number of inappropriate antibiotics prescribed in Tennessee. By identifying populations that are more likely to be prescribed antibiotics inappropriately, interventions can be more effectively targeted. Efforts to reduce inappropriate prescribing should focus on enhancing healthcare provider education about antibiotic stewardship, particularly in outpatient settings. Public health campaigns aimed at educating both patients and providers about the risks of overprescribing antibiotics could further reduce unnecessary prescriptions.

## 4. Materials and Methods

TDH performed a cross-sectional study to assess health disparities in antibiotics prescribing in Tennessee. The IQVIA Longitudinal Prescription Claims (LRx) database tracks more than 90% of retail pharmacy prescriptions, detailing medication names, dosages, dosing frequencies, and the duration of supply. The IQVIA Medical Claims (Dx) database provides outpatient medical claims data, encompassing all International Classification of Diseases, Tenth Revision, Clinical Modification (ICD-10-CM) diagnosis codes assigned by healthcare providers during clinical visits. These data are sourced from health insurers, medical record systems, and coding facilities. TDH obtained and merged IQVIA LRx/Dx data from 1 January 2022 to 31 December 2022. Using a deidentified, unique patient identifier with a pharmacy fill date, the Dx database was queried to identify provider visits within the preceding seven days, and all ICD-10 diagnoses codes from those visits were identified.

We followed the methodology previously published by Fleming-Dutra et al. and applied an antibiotic tier to all ICD-10 diagnosis codes [2]. Additionally, we had two infectious disease physicians independently validate the tier system. Tier 1 diagnoses typically necessitate antibiotic treatment (e.g., pneumonia due to *Staphylococcus aureus*), Tier 2 diagnoses may occasionally require antibiotics (e.g., acute pharyngitis), and Tier 3 diagnoses rarely, if ever, warrant antibiotic use (e.g., viral respiratory illness). If multiple diagnosis codes were present, the higher tier was assigned. For example, if a prescription had ICD-10 codes for both the acute nasopharyngitis (J00, Tier 3) AND other specified bacterial agents as the cause of diseases classified elsewhere (B96, Tier 1), it would be considered Tier 1. Lastly, for the purposes of this analysis, Tier 1 and Tier 2 were categorized as “appropriate” and Tier 3 was categorized as “inappropriate”.

After categorizing each antibiotic into this tiered system, we used descriptive statistics to characterize each tier. The variables that were available and included in this analysis were patient age group, gender, insurance type, and the CDC Social Vulnerability Index (SVI) of the pharmacy zip code using the 2020 U.S. Census Bureau information. Insurance type was defined as the insurance used for the prescription claim. The SVI is a composite score of 16 U.S. Census variables, including but not limited to poverty, access to transportation, and crowded housing, that indicates the potential degree of adversity a community experiences due to external stressors. The closer to one, the more socially vulnerable a population is considered. Conversely, patients living in zip codes with an SVI closer to zero had less social vulnerability. We subsequently used the following quartiles for analysis: 0 to 0.2500 (low), 0.2501 to 0.5000 (low-medium), 0.5001 to 0.7500 (medium-high), and 0.7501 to 1.000 (high). To best approximate patient residence, we used the zip code of the pharmacy where the prescription was filled for SVI. A multivariable logistic regression model was used to predict the factors associated with appropriate antibiotic prescriptions. Two-sided *p* < 0.05 indicated statistical significance. Data were analyzed using SAS software, version 9.4 (SAS Institute Inc., Cary, NC, USA). 

## 5. Conclusions

This study highlights the critical role of demographic and socioeconomic factors in shaping antibiotic prescribing patterns in Tennessee. By identifying specific populations and regions where overprescription is more likely, targeted interventions can be developed to reduce inappropriate antibiotic use and improve healthcare outcomes. Ultimately, addressing these health inequities through data-driven public health strategies will help to prevent the rise in antimicrobial resistance, improve the quality of care, and promote health equity.

## Figures and Tables

**Table 1:** Overall demographic data.

2022 IQVIA Data (n = 2,874,505)							
		Appropriate				Inappropriate	
	Total	Tier 1		Tier 2		Tier 3	
		**N**	**%**	**N**	**%**	**N**	**%**
N (%)	2,874,505	385,008	13.4%	784,020	27.3%	1,705,477	59.3%
Sex							
Male	1,097,942	120,376	11.0%	319,705	29.1%	657,861	59.9%
Female	1,776,563	64,632	14.9%	464,315	26.1%	1,047,616	59.0%
Age							
0–2	77,518	2430	3.1%	46,989	60.6%	28,099	36.2%
3 to 9	246,298	12,366	5.0%	146,216	59.4%	87,716	35.6%
10 to 19	191,165	12,846	6.7%	97,314	50.9%	81,005	42.4%
20 to 29	206,270	35,440	17.2%	70,591	34.2%	100,239	48.6%
30 to 39	224,323	30,624	13.7%	70,333	31.4%	123,366	55.0%
40 to 64	838,856	109,119	13.0%	194,079	23.1%	535,658	63.9%
65 and older	1,090,075	182,183	16.7%	158,498	14.5%	749,394	68.7%
SVI							
Low	823,019	113,074	13.7%	230,815	28.0%	479,130	58.2%
Low-medium	697,206	92,146	13.2%	188,621	27.1%	416,439	59.7%
Medium-high	650,666	88,689	13.6%	171,546	26.4%	390,431	60.0%
High	703,704	91,099	12.9%	193,038	27.4%	419,567	59.6%
Insurance Type							
Missing	44	3	6.8%	10	22.7%	31	70.5%
Cash	48,840	7695	15.8%	13,172	27.0%	27,973	57.3%
Medicaid	561,613	63,304	11.3%	217,186	38.7%	281,123	50.1%
Third Party	1,257,276	149,247	11.9%	405,029	32.2%	703,000	55.9%
Medicare Part D	1,006,732	164,759	16.4%	148,623	14.8%	693,350	68.9%

**Table 2:** Multivariate analysis predicting inappropriate antibiotic prescriptions.

	OR (95% CI)	*p*-Value ^a^
Gender		
Female	0.89 (0.89, 0.89)	<0.001
Male ^Ref^	-	-
Age		
0–2	0.27 (0.26, 0.27)	<0.001
3 to 9	0.35 (0.35, 0.35)	<0.001
10 to 19	0.47 (0.46, 0.47)	<0.001
20 to 29	0.26 (0.26, 0.26)	<0.001
30 to 39	0.60 (0.60, 0.61)	<0.001
40 to 64	0.87 (0.86, 0.87)	<0.001
65 and older ^Ref^	-	-
Social Vulnerability Index		
Low	0.94 (0.94, 0.95)	<0.001
Low-medium	1.00 (0.99, 1.00)	0.21
Medium-high	1.00 (0.99, 1.00)	0.14
High ^Ref^	-	-
Insurance Type		
Cash	0.98 (0.97, 1.00)	0.08
Medicaid	1.17 (1.16, 1.18)	<0.001
Medicare Part D	1.20 (1.20, 1.21)	<0.001
Third Party ^Ref^	-	-

^a^ *p*-values < 0.05 were considered statistically significant. ^Ref^ reference.

## Data Availability

Data are available upon request through a standard data access request form from the Tennessee Department of Health. The Department complies with all applicable U.S. Federal and Tennessee laws regarding data access.
